# Quantitative Blood Flow Measurements in the Common Carotid Artery: A Comparative Study of High-Frame-Rate Ultrasound Vector Flow Imaging, Pulsed Wave Doppler, and Phase Contrast Magnetic Resonance Imaging

**DOI:** 10.3390/diagnostics12030690

**Published:** 2022-03-11

**Authors:** Yigang Du, Haiyan Ding, Le He, Billy Y. S. Yiu, Linsong Deng, Alfred C. H. Yu, Lei Zhu

**Affiliations:** 1 Shenzhen Mindray Bio-Medical Electronics Co., Ltd., Shenzhen 518057, China; duyigang@mindray.com (Y.D.); denglinsong@mindray.com (L.D.); 2 Department of Biomedical Engineering, Tsinghua University, Beijing 100084, China; dinghy@mail.tsinghua.edu.cn (H.D.); hele0806@aliyun.com (L.H.); 3 Schlegel Research Institute for Aging, University of Waterloo, Waterloo, ON N2L 3G1, Canada; billy.yiu@uwaterloo.ca (B.Y.S.Y.); alfred.yu@uwaterloo.ca (A.C.H.Y.)

**Keywords:** vector flow imaging, PW, PC-MRI, velocities and volume flow measurements

## Abstract

V Flow is commercially developed by high-frame-rate ultrasound vector flow imaging. Compared to conventional color Doppler, V Flow is angle-independent and is capable of measuring both the magnitude and the direction of blood flow velocities. This paper aims to investigate the differences between V Flow and pulsed wave Doppler (PW) relative to phase contrast magnetic resonance imaging (PC-MRI), for the quantitative measurements of blood flow in common carotid arteries (CCA) and, consequently, to evaluate the accuracy of the new technique, V Flow. Sixty-four CCAs were measured using V Flow, PW, and PC-MRI. The maximum velocities, time-averaged mean (TAMEAN) velocities, and volume flow were measured using different imaging technologies. The mean error with standard deviation (Std), the median of absolute errors, and the r-values between V Flow and PC-MRI results for the maximum velocity, the TAMEAN velocity, and the volume flow measurements are {9.40% ± 14.91%; 11.84%; 0.84}, {21.52% ± 14.46%; 19.28%; 0.86}, and {−2.80% ± 14.01%; 10.38%; 0.7}, respectively, and are {53.44% ± 29.68%; 49.79%; 0.74}, {27.83% ± 31.60%; 23.83; 0.71}, and {21.01% ± 29.64%; 25.48%; 0.34}, respectively, for those between PW and PC-MRI. The boxplot, linear regression and Bland–Altman plots were performed for each comparison, which illustrated that the results measured via V Flow rather than via PW agreed more closely with those measured via PC-MRI.

## 1. Introduction

Doppler ultrasound is one of the most commonly used medical imaging techniques for the routine examination of vascular disease. In clinics, carotid artery stenosis can be diagnosed using ultrasonography (US) via B-mode grayscale images and the peak systolic (PS) and end-diastolic (ED) flow velocities, which are the maximum values at the location (along the vessel diameter), identified visually using color Doppler, and measured quantitatively by pulsed wave Doppler (PW). The degree of stenosis for carotid arteries can be detected and quantified by measuring the narrowest diameter of the remaining stenotic lumen directly and by comparing it to the distal normal lumen (NASCET [1]) or the estimated original lumen at its narrowest point (ESCT [2]). This could be performed based on the carotid angiography, which is an invasive imaging technique that still has several deficiencies in assessing stenosis, in combination with the above methods (NASCET and ESCT). Firstly, the entire intimal thickening of the proximal and distal arteries may not be reflected by the NASCET method [3]. Secondly, it is extremely difficult to measure the original lumen using angiography when the ESCT method is employed [3]. To improve the performance of the diagnosis methods further, the use of Doppler US was proposed. This technique can facilitate the diagnosis of internal carotid artery (ICA) stenosis, using PW to measure the peak systolic and end-diastolic velocities of ICA, as well as the peak systolic velocity ratios between the ICA and the common carotid artery (CCA); these velocities are used to classify ICA stenosis [4,5].

It is essential to make an accurate estimation of blood flow velocities; however, the conventional Doppler US (e.g., PW) is angle-dependent, measuring only one-dimensional velocity components along the ultrasound beam, and the actual flow angle has to be corrected according to the shape of the blood vessels, assuming that the blood travels parallel to the vessel wall [6]. This assumption is appropriate for extremely long and straight blood vessels [7]; however, it does not make sense in curved arteries [8] and is definitely incorrect in stenoses (diseased vessels) or bifurcations [9]. Moreover, the angle correction of PW is usually operated manually, generating uncertain errors of velocity estimation, which leads to the inaccurate diagnosis of artery stenosis [3]. The error associated with the velocity estimation increases by 20~30% even for a 5° error in angle correction, if the beam-to-flow angle cannot be kept below 60° [10]. 

Compared to conventional Doppler US, vector flow imaging (VFI) is an innovative technology [11], in which both the magnitude and the direction of a true velocity on the imaging plane can be obtained (note that, in this paper, VFI denotes all techniques providing vector velocities in the 2D imaging plane). The angle-independent technique had been proposed many years ago [12], with different methods developed to realize it successfully [13,14], including, but not limited to, speckle tracking [12,15], transverse oscillation (TO) [16], fluid continuity solved via boundary conditions [17], multi-directional Doppler transmission and/or reception [18,19], and contrast-enhanced VFI [20]. Unlike PW, there are still no clear or specific diagnostic criteria based on the velocity measurement of VFI in clinics. This implies that the VFI results are yet to be accepted completely in clinical environments, possibly due to the different implementations of VFI producing different results. In addition, the function of current commercially available VFI systems is restricted to the 2D imaging plane; therefore, they do not reflect a physiological 3D flow measurement.

For the clinical application of quantitative results-based diagnosis, both the accuracy and reproducibility of the VFI-based measurements must be prioritized. Previously, TO-based VFI has been developed for industry, with measurements validated by magnetic resonance angiography (MRA) [21,22]. In these studies, the stroke volume [21] and vector velocity [22] are compared to the MRA results. These comparisons show that volume flow results for TO are closer to MRA (r = 0.91) than other VFI-based techniques, i.e., directional beamforming (DB, r = 0.84) and synthetic aperture flow imaging (STA, r = 0.71) [21]. Furthermore, for velocity estimation, TO (error: 18%~24%) provides a more accurate estimation, relative to MRA, than PW (error: 23~38%) [22]. More recently, a new tool for the evaluation of complex flow, known as V Flow, which performs high-frame-rate dynamic visualization VFI [19], has been implemented for a clinical ultrasound system [23]. The V Flow technique is based on multi-directional Doppler US [19] using an interleaved plane wave and focused wave transmissions [24]. Therefore, the flow signals of V Flow are completely different from the TO-based VFI presented in [22]. In general, multi-directional Doppler increases the sensitivity of V Flow, which has a much higher temporal resolution due to its ultrafast imaging capability; however, it sacrifices the signal-to-noise ratio (SNR) and blood-to-noise ratio (BNR) compared to the TO technique, since it uses focused beams for VFI. This study aims to evaluate the accuracy of velocity and volume flow measurements for the high-frame-rate V Flow function. Phase contrast magnetic resonance imaging (PC-MRI) was used as a reference imaging technique. The flow measurements of CCAs were performed using V Flow with conventional PW ultrasound, and were compared to the results measured via PC-MRI.

The rest of this paper is structured as follows. Section 2 introduces the imaging techniques and scanning setups used to conduct flow measurements, and presents a scheme of the techniques being compared. Section 3 presents the measurement results, which are discussed in terms of the differences among the compared techniques in Section 4. The conclusion is given in the last section.

## 2. Materials and Methods

This study was approved by the local Institutional Review Board of Tsinghua University. Written informed consent was obtained from all subjects. Two ultrasound techniques and PC-MRI were used for the examination of both the left and right common carotid arteries (CCAs) for all presumed healthy volunteers, including 15 males and 17 females, with an average age of 49, ranging from 27 to 65 years old. Thus, 64 CCAs were measured using the three imaging techniques. Due to the wide age range, it was possible to investigate a wide range of flow velocities for the comparison studies. For each participant, the total examination, including PW, V Flow, and PC-MRI, was completed within a 2 h period on the same day (PC-MRI: 0.5 h; PW + V Flow: 0.5 h; the order of the three examinations was randomized). The ultrasound and MRI scans were performed in the supine position and were operated mainly by a sonographer–L.D. (10-years+ experience with ultrasound systems), and an MR operator–L.H. (20-years+ experience with MRI systems), respectively.

### 2.1. V Flow Technique Description

V Flow is a high-frame-rate (FR: 374~1240 Hz) dynamic displayed ultrasound vector flow imaging technique that is commercially available for the Resona 7 ultrasound system. It employs multi-directional ultrasound transmission and reception to generate velocity components along different angles [19,23,25]. Each velocity component is estimated by the Doppler technique using the conventional lag-1 auto-correlation [26]. The Doppler transmission is interleaved with focused waves to generate a high-spatial-resolution grayscale B-mode image simultaneously with the vector flow [24]. The vector velocity is reconstructed based on the estimated velocity components.

### 2.2. Ultrasound Scan Setup

For ultrasound scans, the quantitative blood flow measurements for the common carotid artery (CCA) were made using a linear array transducer (L11-3U) via PW and V Flow using a commercial ultrasound system, Resona 7, manufactured by Shenzhen Mindray Bio-Medical Electronics Co., Ltd. (Shenzhen, China). Both PW and V Flow are commercially released functions on the Resona 7 system. 

**Figure 1:**
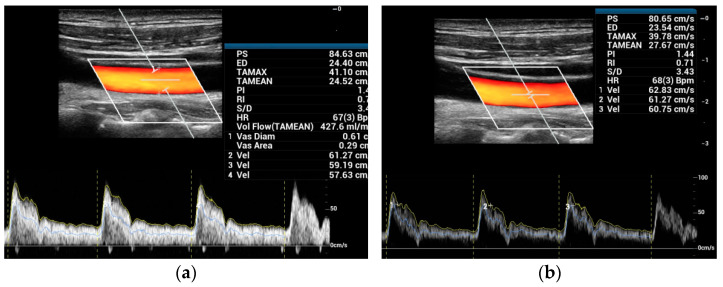
PW measurements: (**a**) big SV covering the vessel for measuring time-averaged mean velocity (TAMEAN) and volume flow (Vol Flow); (**b**) small SV for measuring the maximum velocity (PS).

The blood flow velocity and volume flow of CCA were measured via PW and V Flow. For PW, the results were corrected using a conventional angle correction line, as shown in the example in Figure 1. The peak systolic (PS) and time-averaged mean (TAMEAN) velocities, and volume flow during two or three cardiac cycles, were measured using PW with different SVs. A big SV (4–7.5 mm, Figure 1a) covering the vessel was used for the mean velocity and volume flow measurements, and a small SV (0.5–1.5 mm, Figure 1b) was used to measure the maximum velocity. 

**Figure 2:**
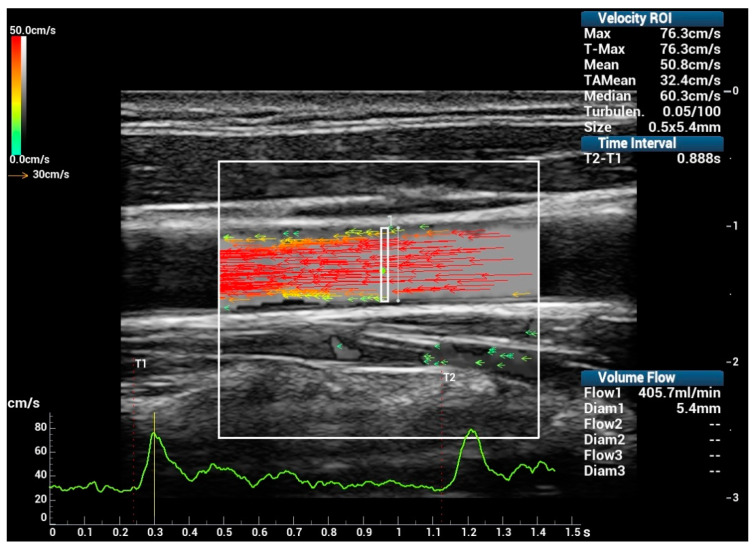
V Flow measurements: long ROI spanning the vessel diameter for measuring the maximum velocity (T-Max), time-averaged mean velocity (TAMean), and volume flow (Flow1).

For V Flow, flow data were acquired continuously for 1.5 s. The frame rate was around 500~600 fps set by users depending on the estimated velocities. The maximum and time-averaged mean velocities (T-Max and TAMean for V Flow) within a long ROI spanning the vessel diameter (the white rectangular box in Figure 2) during one cardiac cycle were measured. The volume flow was measured by integrating all velocity components along the inner diameter of the vessel (the white line in Figure 2), where the velocity components perpendicular to the diameter are derived from the measured vector velocities. The cross-section of the vessel is assumed to be circular and can be divided into several annular areas. The velocity components corresponding to specific annular areas were used to estimate the corresponding volume flow components, and then accumulated to obtain the final volume flow rate. All V Flow data used for the analysis were obtained within one cardiac cycle, selected using “T1” and “T2” (see Figure 2) with the assistance of the velocity curve (i.e., the green curve in Figure 2).

### 2.3. PC-MRI Scan Setup

**Figure 3:**
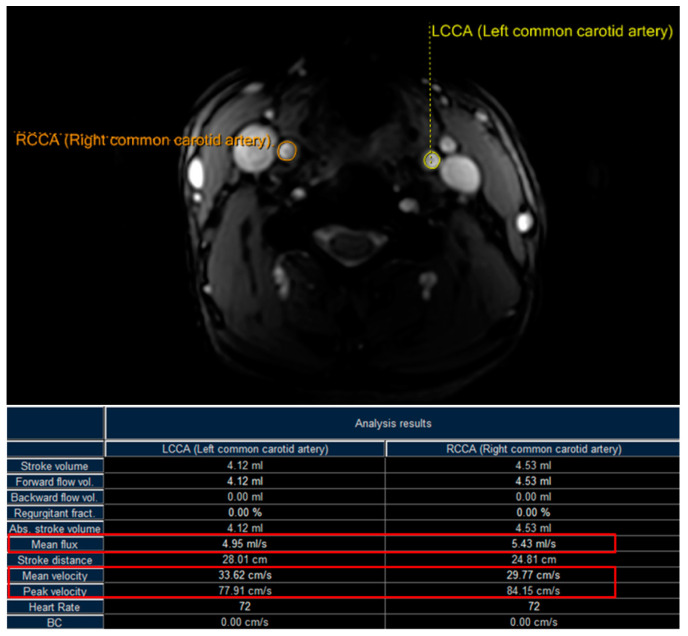
PC-MRI measurements of both the left and right common carotid arteries for the maximum and time-averaged mean velocities, and volume flow rate (“Peak velocity”, “Mean velocity”, and “Mean flux” denoted in the figure).

All MR experiments were performed on a multi-transmit 3T MR scanner (Ingenia CX, Philips Healthcare, Best, Netherlands) using a 20-channel head–neck coil. The general acquisition parameters for 2D PC-MRI of each CCA were: FOV—150 by 150 mm^2^; imaging resolution—1.17 by 1.17 mm^2^, reconstructed into 0.59 by 0.59 mm^2^; slice thickness—5 mm; spoiled gradient echo (SPGR) as readout with flip angle—10°; and TR/TE—13.0/7.9 ms. The phase contrast flow direction was the foot–head direction with a velocity encoding (VENC) of 90 cm/s. The peripheral pulse unit (PPU) was used to synchronize the scan with 15 heart phases. The maximum and time-averaged mean velocities and the volume flow rate are estimated for both the left and right common carotid arteries (a total of 64 CCAs). One example is shown in Figure 3. Results from 3 CCAs had to be abandoned due to aliasing (i.e., the real velocity being larger than the detectable 90 cm/s). Therefore, the PC-MRI results for 61 CCAs are used in the comparison studies.

### 2.4. Statistical Analysis

The PC-MRI measurements were used as the reference (benchmark). The data were processed for comparison studies by Matlab (The MathWorks, Natick, MA, USA), which was used to calculate all statistical results and to generate plots. The results were obtained using PW and V Flow for the maximum and TAMEAN velocities, and the volume flows were compared to those obtained using PC-MRI. The mean error with standard deviation and the median of absolute errors were calculated. The boxplot was performed based on the errors relative to PC-MRI results for PW and V Flow. The Pearson correlation coefficient (r-value) was calculated for each comparison to study the correlation and similarity of the results measured via different imaging techniques. The linear regression and Bland–Altman plots were also used to illustrate the differences for each comparison.

## 3. Results

**Figure 4:**
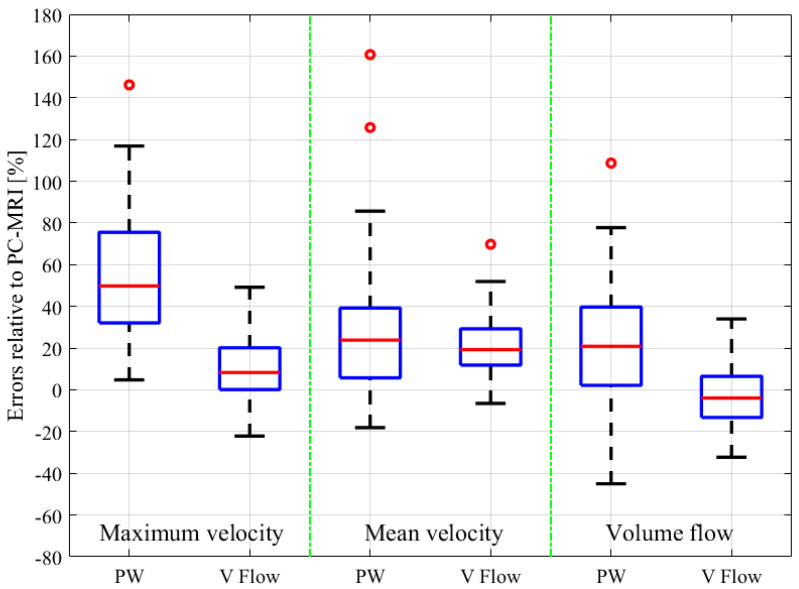
Boxplot for the relative errors of PW and V Flow compared to PC-MRI for maximum velocity, mean velocity and volume flow measurements.

**Figure 5:**
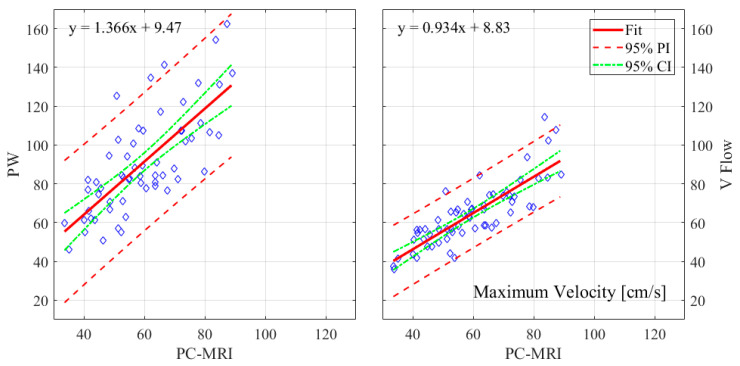
Linear regression plots of maximum velocities with 95% PI and 95% CI for PW and V Flow relative to PC-MRI.

**Figure 6:**
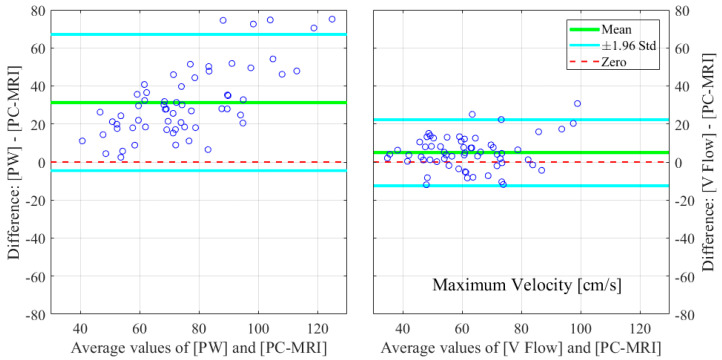
Bland–Altman plots for illustrating the differences in the estimated maximum velocities for PW and V Flow relative to PC-MRI.

**Figure 7:**
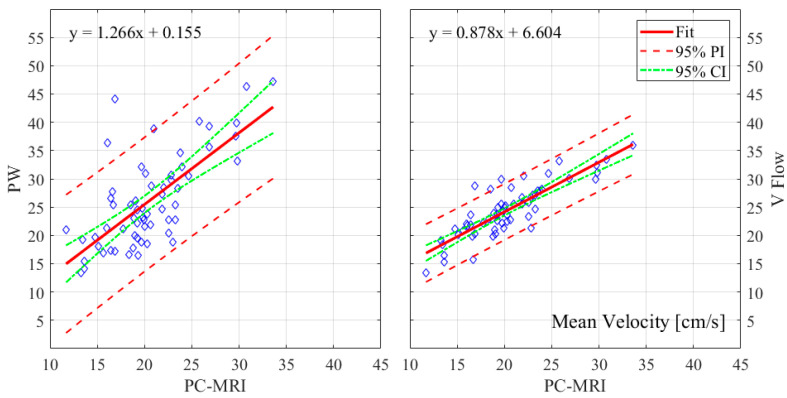
Linear regression plots of mean velocities with 95% PI and 95% CI for PW and V Flow relative to PC-MRI.

**Figure 8:**
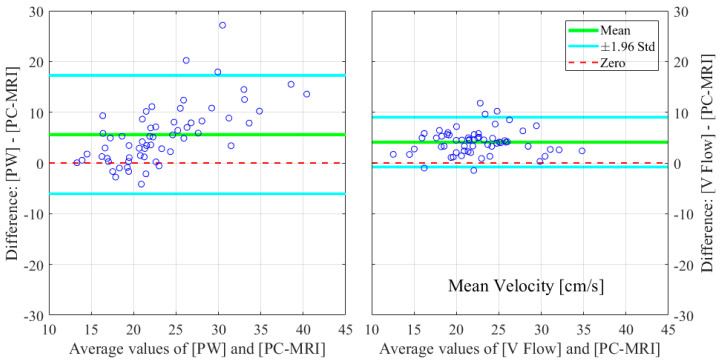
Bland–Altman plots for illustrating the differences in the estimated mean velocities for PW and V Flow relative to PC-MRI.

**Figure 9:**
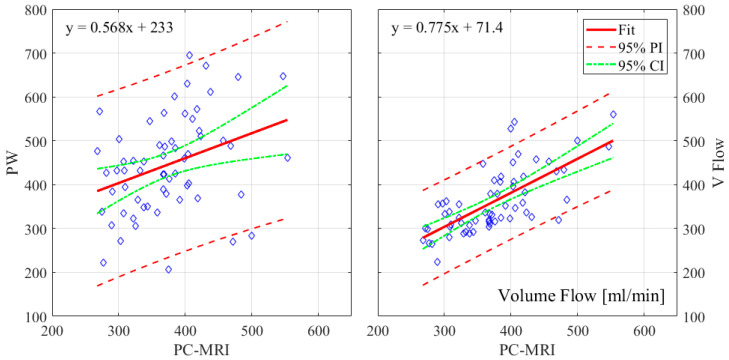
Linear regression plots of volume flow measurements with 95% PI and 95% CI for PW and V Flow relative to PC-MRI.

**Figure 10:**
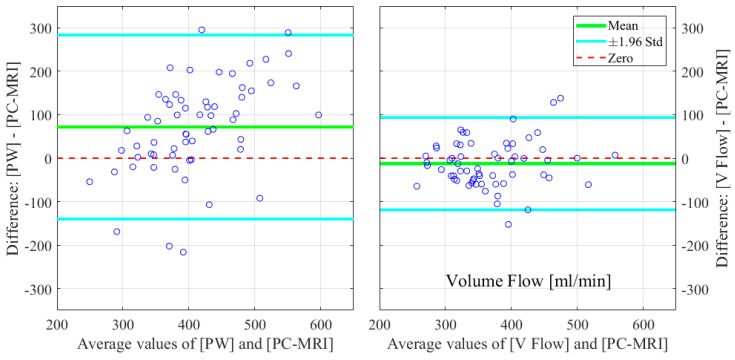
Bland–Altman plots for illustrating the differences in the estimated volume flow for PW and V Flow relative to PC-MRI.

The relative errors and r-values of V Flow and PW compared to the PC-MRI measurements are listed in Table 1. As described in Section 2.3, results from three CCAs for PC-MRI measurements had aliasing and, thus, were not included. The comparisons were performed for 61 CCA measurements, with the exception of the maximum velocity for PW, since the recording of one of these results was missed during the examination; hence, 60 CCAs were compared for this. The corresponding boxplot (PW or V Flow vs PC-MRI) is shown in Figure 4, where *p*-value = 0.07 (differences were considered statistically significant at *p* < 0.05) for the volume flow between V Flow and PC-MRI measurements, and *p*-value < 0.001 for the rest of the compared results. The linear regression and Bland–Altman plots are shown in Figure 5 and Figure 6 for the maximum velocity estimations, in Figure 7 and Figure 8 for the mean velocity estimations, and in Figure 9 and Figure 10 for the volume flow estimations. In the linear regression plots, the fitted line, 95% prediction intervals (PI), and 95% confidence intervals (CI) are displayed. In the Bland–Altman plots, the green line denotes the mean bias and the light blue lines denote the limits of agreement. Evidently, all the comparison plots show that the V Flow results are more reliable and closer to the measured values of PC-MRI than PW.

## 4. Discussion

All measurements obtained by the V Flow technique have better agreement and correlations with PC-MRI results, compared to those by PW (Table 1). Significant differences between the conventional PW and PC-MRI measurements of the maximum velocity, mean velocity, and volume flow (*p*-values < 0.001) can be found in this comparison study. From the boxplot, regression, and Bland–Altman plots, it can be seen that the velocity results are clearly overestimated by PW relative to PC-MRI. For V Flow, the limits of agreement contain the zero line, which means the bias is not statistically different from zero (i.e., no systemic bias). The spreads of the V Flow vs. PC-MRI measurements are clearly smaller than those of PW vs. PC-MRI, which also have more data points outside the limits of agreement (Figure 5, Figure 6, Figure 7, Figure 8, Figure 9 and Figure 10). 

The overestimation of the maximum velocity for PW is caused mainly by the “geometric spectral broadening” effect, whereby the true angles of the bilateral arrays generating and receiving the PW Doppler beam are different from the assumed Doppler angle along the aperture center [27,28]. This broadens the Doppler signal (spectrum), even if the red blood cells (RBCs) in SV only have a single velocity. Moreover, the velocity, during the period (5~40 ms) of the PW calculation, may change significantly [29]; in the SV, there are millions of RBCs, each with a different velocity, resulting in a broad spectrum, where the values from the envelope curve are probably larger than those produced by other techniques that use averaging curves.

For the mean velocity, the results are overestimated by both PW and V Flow measurements, relative to PC-MRI. In Figure 4, Figure 7 and Figure 8, it can be seen that the average values are clearly higher than the zero levels. The overestimation can be explained by the following aspect: the mean velocity is measured based on the velocities of RBCs along the diameter for PW and V Flow, whereas it is based on all of the RBC velocities through the cross-sectional area for PC-MRI. This difference means that more RBCs with small velocities around the margin of the cross-section are included to obtain the mean velocity for PC-MRI measurements. This phenomenon has been simulated in [30], which presents a difference as 0.66 cm/s vs. 0.49 cm/s for the diameter-based and cross-section-based mean velocity estimations.

For the volume flow measurements, different compared results are shown for PW and V Flow. Significant differences (*p*-value < 0.001) and overestimation (Figure 4, Figure 9 and Figure 10) can still be found for PW; however, none of these are found for V Flow. This is because the estimating methods of volume flow for PW and V Flow are different. For PW, it is completely based on the mean velocity (TAMEAN) and the diameter of the vessel. Therefore, the volume flow is overestimated since the mean velocity is overestimated. Although more RBCs with low velocities around the cross-section could be included for PC-MRI measurements, they are considered too low to contribute. For V Flow, as presented in the previous paragraph, the velocity components perpendicular to the diameter are considered, which generates a set of velocity components along the diameter, and their average values must be lower than the previously measured mean velocity. Therefore, the volume flow measured via V Flow has a lower result compared to PW. Moreover, smaller errors and a much better correlation between V Flow and PC-MRI results are obtained for volume flow than those between PW and PC-MRI (Table 1).

In previous studies, the TO-based VFI emerged with a higher precision of velocity measurement than PW at different examination sites [22]. As a high-frame-rate, multi-directional, Doppler-based VFI, V Flow also performed better than PW in both velocity and volume flow measurements. However, neither V Flow nor PW are spatially 3D imaging techniques, in that V Flow is restricted to the estimation of two-dimensional vector velocities on the imaging plane, while PW is only for measuring one-dimensional velocity. Therefore, out-of-plane velocities cannot be measured by V Flow. This could cause some errors if the imaging plane is not exactly on-axis (longitudinal axis of the vessel), or if spiral/helical and secondary flows exist, which can only be detected in short-axis view via a 2D VFI [31,32,33] or a full 3D VFI [34,35]. The turbulent or more complex flows found at bifurcations, in irregular vessels, or in very stenotic vessels with complicated shapes require further investigation with computational fluid dynamics (CFD) in another work. Moreover, in addition to velocity and volume flow measurements, the resistance index, and the pulsatile index, are also essential parameters of hemodynamics, and their accuracy (V Flow vs PC- MRI) will be studied in future work.

## 5. Conclusions

Overall, the estimating results of V Flow, rather than PW, have a better agreement with the PC-MRI measurements. The velocities are overestimated by both PW and V Flow compared to PC-MRI estimations, but with no systemic bias for V Flow. The volume flow is overestimated by PW, but with no significant difference for the volume flow of the V Flow estimation compared to PC-MRI. High-frame-rate vector flow imaging based on multi-directional Doppler transmission and reception is a promising technology that achieves more accurate quantitative measurements for the CCA compared to conventional PW.

## Figures and Tables

**Table 1:** The mean error with standard deviation (Std), the median of absolute errors, and the r-value of V Flow and PW relative to the PC-MRI results.

Error [%]: Mean ± Std	Maximum Velocity	Mean Velocity	Volume Flow
PW	53.44 ± 29.68	27.83 ± 31.60	21.01 ± 29.64
V Flow	9.40 ± 14.91	21.52 ± 14.46	−2.80 ± 14.01
Error [%]: Median	Maximum Velocity	Mean Velocity	Volume Flow
PW	49.79	23.83	25.48
V Flow	11.84	19.28	10.38
r-Value (no. of Vessels)	Maximum Velocity	Mean Velocity	Volume Flow
PW	0.74 (60 CCAs)	0.71 (61 CCAs)	0.34 (61 CCAs)
V Flow	0.84 (61 CCAs)	0.86 (61 CCAs)	0.7 (61 CCAs)

## Data Availability

Clinical data are available from the corresponding author upon reasonable request.

## List of Abbreviations

VFI: vector flow imaging; PW: pulsed wave; PC-MRI: phase contrast magnetic resonance imaging; MRA: magnetic resonance angiography; CCA: common carotid artery; ICA: internal carotid artery; US: ultrasonography; PS: peak systolic; ED: end-diastolic; NASCET: North American Symptomatic Carotid Endarterectomy; ESCT: European Carotid Surgery Trial; SNR: signal-to-noise ratio; BNR: blood-to-noise ratio; TO: transverse oscillation; DB: directional beamforming; STA: synthetic aperture flow imaging; SV: sample volume; Std: standard deviation; ROI: region of interest; TAMEAN/TAMean: time averaged mean; PI: prediction interval; CI: confidence interval; RBC: red blood cell; CFD: Computational Fluid Dynamics.
